# Meaning of Success: perception of medical students, and faculty-A Qualitative Study from a medical school in Mauritius

**DOI:** 10.3126/nje.v10i3.28424

**Published:** 2020-09-30

**Authors:** Indrajit Banerjee, Jared Robinson, Bhavna Munoosingh, Nidhi Jain, Ramya S Amsadevi

**Affiliations:** 1 Department of Pharmacology, Sir Seewoosagur Ramgoolam Medical College, Belle Rive, Mauritius; 2 Sir Seewoosagur Ramgoolam Medical College, Belle Rive, Mauritius; 3 Sir Seewoosagur Ramgoolam Medical College, Belle Rive, Mauritius; 4 Sir Seewoosagur Ramgoolam Medical College, Belle Rive, Mauritius; 5 Sir Seewoosagur Ramgoolam Medical College, Belle Rive, Mauritius

**Keywords:** Achievement, Hermeneutics, Empirical Research, Psychology, Educational, Indian Ocean Islands

## Abstract

**Background:**

The objective of this study was to find what undergraduate medical students and teaching faculty perceive success to be.

**Methods:**

A descriptive phenomenological qualitative study was designed and conducted on faculty and medical students in Sir Seewoosagur Ramgoolam Medical College, Mauritius. NVivo 12 (Windows) Plus software was implemented for data analysis and thematic analysis was performed.

**Results:**

The codes/nodes namely being: Satisfaction, Accomplishment, Actions, Motivations, Extrinsic Factors and Intrinsic Factors were identified in the transcribed data. Satisfaction was described as the positive emotions and notions intimately related as well as synonymously associated with success. Accomplishment as the attainment and fulfilment of any physical, mental, emotional, social, occupational, personal goal or desire by an individual. Actions was the arsenal of physical processes, acts of planning, goal setting or forethinking exercised by the individual. Motivations was the drive to attain the preset goal or notion be it positive or negative. This applies to factors that enable a subject to strive forwards. Extrinsic Factors were the external determinants and definition of success perceived by the subject. Intrinsic Factors were the subject’s internal organic, comprehension and definition of success. The themes generated were: Products of Success, Mechanisms of Success and Concepts of success.

**Conclusion:**

A tangible demarcation is noticeable between the preconceived general impression of success and the vast multifactorial cohort of intrinsic and extrinsic factors coupled to the highly emotional aspects which were brought forth.

## Introduction

Success is a concept which is ever changing and has countless as well as unfathomable depths of perceptions, notions, comprehensions and definitions. The manner and light in which one views, interprets and defines success, showcases an individual’s true beliefs and grounding as well as lends insight into how that individual perceives the world. Since the dawn of civilization, man has strived to meet his basic needs: food, shelter and clothing. However, over the course of time one’s needs have increasingly been influenced by their wants, which have been one of the everlasting sources and roots of human happiness [1].

On this planet driven by competition, the desire to succeed has become of the utmost significance [2]. As a result, thereof, the relevance of an individual human life is now being increasingly gauged on one’s ability to succeed in one’s lifetime [3,4]. However, when it comes to understanding the perception of success within society. It became evident that success is principally a highly multifaceted and individualistic concept [5]. During this research, many articles were studied to derive the general perception of success [6].

In terms of the medical sphere a study conducted by R. Jagsi et al. in 2017 surveyed clinician-researchers who were awarded with K08 and K23 awards. This study providing the perception that success in the medical field can be measured by and is about producing award winning research. Further insights provided were that a host of factors played a role in a clinician’s ability to produce such research and hence become successful namely: the gender of the researcher, demographics, work atmosphere, job features, and domestic commitments [7].

A study conducted by Angela L Duckworth and team on 9,646 American adults based their research on success via dividing the attainment thereof into two categories namely in the objective realm as well as the subjective realm. This study acting as a baseline for the division of success. The study further concluded that adults who were conscientious in nature were more likely to excel in both of the realms [8]. The study conducted subdivides success into the two broad based categories of namely objective and subjective success. This will act as a base on which this study will be built. Although the categorization of success acts as a base it does not define and compartmentalize success to a degree where the meaning and understanding thereof is clearly elucidated.

Upon having asked Warren Buffet about his opinion on success, Bill Gates was awed by the former’s answer on how his loved ones’ happiness and the love he derived from them were the sole things which made him happy. On his end, Bill Gates emphasized on how success would be incomplete without invention for the purpose of helping people in need and for raising children in a holistic environment [9]. Success acts as a universal gateway as it delineates no race nor creed. It is a concept of universal understanding. The further expansion on previous studies thus has the potential for the full mechanism and understanding thereof to be truly attained and plotted. It is essential for this concept to be fully explored.

This research was carried out with the primary aim of understanding the true meaning of success within faculty and medical students at Sir Seewoosagur Ramgoolam Medical College (SSR Medical College). However, it is empirical to also understand as to why success is such an essential component for human life. Understanding success and its complexities is vital as success has an impact on our mental wellbeing and psychology. Success aids an individual in understanding life and it lays down the basis of setting aims and plans [10]. The objective of this study was to find what undergraduate medical students and teaching faculty perceive success to be.

## Methodology

### Study design and participants

A descriptive phenomenological qualitative study was designed and was conducted on a total of ten faculty members and ten undergraduate medical students at SSR Medical College, Mauritius. The participation in the study was voluntary, and written consent was taken from each of the study participants. The data was collected on a in depth one on one interview basis and was recorded.

### Data Collection

The study was conducted from the 15th August to the 5th September 2019 at SSR Medical College, Mauritius [11]. The interviews were conducted by the authors and the recorded interview data was transcribed by the authors using both voice to text translation applications and manual transcription methods, the transcribed text was ultimately vetted and corrected by manual transcription of the recorded audio on a word for word basis. Each of the authors cross checked one another’s transcribed interviews to ensure both accuracy and quality thereof. The completed transcripts were printed and participants were allowed to verify the text. Once the text was verified all of the recordings and data was transferred to an encrypted device and stored to ensure sanctity and confidentiality of the data. Each individual candidate that partook in this study, was first given an explanation as to how their data would be used as well as was ensured about the process of the data collection. Candidates were also made aware that their anonymity would be maintained. Every candidate was then asked whilst being voice recording whether they would give an informed consent for this study. The members which did not give an informed consent were not interviewed and the interview was subsequently terminated. The participants reserved the right to not answer certain questions, as well as maintained the right to retract any statements or abstain from being part of the study. Participants were also free to exercise their right to withdraw from the study at any point in time if they so wished.

#### Questionnaire design and validation

A semi-structured open-ended questionnaire was formulated to conduct the interview. The questionnaire was validated by three experts. A pilot study of five students and two faculty was performed and the results were discussed among the authors to modify the questionnaire. This modified questionnaire was used in the primary study. The same questions were posed to all of the participants.

#### Inclusion criteria

To ensure valid and equal participation and representation of members of SSR Medical College a purposive sampling technique was exercised. A total of ten faculty members and ten students were selected based on age, nationality and year of study to ensure that the data collected best framed the College. One on one in depth interviews were conducted till sample saturation was attained.

#### Exclusion criteria

The involvement in this study was completely voluntary. The students and faculty of SSRMC who were unwilling to participate in the interview and in the study were excluded from the study. Faculty members from the same department were excluded, and similar exclusion parameters were exercised in the year of study.

#### Sample size calculation

The study was a qualitative study, conducted on a total of ten faculty members and ten undergraduate medical students. The data was collected according to the guidelines of Glaser BG et al. untill sample saturation was attained, where no new codes/nodes were generated upon interview[12].

#### Outcome variable

Success was the outcome variable

#### Explanatory variable

The explanatory variables were the medical students and the faculty members at SSR Medical College, Mauritius.

#### Ethical committee approval

The Research was conducted as per the latest version of the Declaration of Helsinki, 64th World Medical Association, General Assembly, Fortaleza, Brazil, October 2013, Helsinki - Ethical Principles for Medical Research involving Human Subjects guidelines [13]. Ethical committee approval was taken from the institutional ethical and review board prior to conducting the study.

#### Data management and statistical analysis

The data was transcribed by the authors (JR, NJ, BM) using both voice to text translation applications and manual transcription methods, the transcribed text was ultimately vetted and corrected by manual transcription of the recorded audio on a word for word basis. The completed transcripts were printed and participants were allowed to verify the text. The iterative process consisted of authors analyzing every transcript in isolation and generating separate individual nodes. IB supervised the data collection and analyzed the data using the NVivo 12 (Windows) Plus software to generate the nodes/codes and the various themes. Once this phase was completed all the authors codes/nodes were compiled and meetings were held to refine the compilation. The codes/nodes underwent analysis and a large degree of semblance was evident. Duplicate codes/nodes were removed and others merged. For codes/nodes to be generated or removed complete accord of the authors was needed. A code/node book was generated, on completion of the coding the authors deduced meaning themes. After much deliberation three themes were determined. The codes/nodes were generated using a latent approach and from the codes/nodes the authors through the above intensive iterative process used a deductive approach to determine the various themes by codebook framework approach. The data was analyzed in accordance to Braun and Clarke’s Six Phases of Thematic Analysis [14].

## Results

### Demographic details:

Demographic details were recorded before conducting the interview. A total number of twenty interviews were conducted, (n=20) which consists of 10(50) teaching faculty were labelled as participant F1-F10 and 10(50) undergraduate medical students were labelled as participant M1-M10 respectively. The mean age of the faculty was found to be 50.10±17.572 years SD and for students 20.80±2.201 years SD respectively. Among the 10 faculty members 6(60) were Indian and 4(40) were Mauritian. Among the medical students 4(40) were Mauritian, 3(30) were Indian and South African respectively. 3(30) students each were from second and final part II professionals, whereas 2(20) students each were selected from first and final part I respectively (Table 1).

In this study subsequent to much analysis and excogitation, six codes/nodes were identified in the raw transcribed data. The codes/nodes can be identified in table 2. The codes/nodes are namely: 1) Satisfaction, 2) Accomplishment, 3) Actions, 4) Motivations, 5) Extrinsic Factors and 6) Intrinsic Factors.

### Satisfaction

“laughing and having a good time”

-Participant F4, Faculty, Male

Satisfaction was described by the data as: The positive emotions and notions intimately related as well as synonymously associated with success.

### Accomplishment

“When I was a commanding officer in the army my job was to become professor of physiology because that was my subject and then if I rise to that level in my subject then I will feel great, and naturally that was success and achievement”

- Participant F6, Faculty, Male

Accomplishment was described by the data as: The attainment and fulfilment of any physical, mental, emotional, social, occupational, personal goal or desire by an individual.

The two above codes allowed for the generation of the theme “Products of Success.” This theme thereby establishing the end goal and aiding as a guideline to certify the true attainment of success by encapsulating both the physical and emotional aspect thereof.

### Actions

“you keep aspiring and moving in that direction”

-Participant F3, Faculty, Male

Actions was described by the data as: The arsenal of physical processes, acts of planning, goal setting or forethinking exercised by the individual.

### Motivations

I think perseverance and self-belief because it is self-belief and self-confidence which gives you positive thinking and if you have positive thinking you will catch onto the opportunity which comes your way and that is what will lead to success.”

- Participant F10, Faculty, Female

Motivations was described by the data as: Drive to attain the preset goal or notion be it positive or negative, applies to factors that enable a subject to strive forwards. Whether it be financial, altruistic, personal familial or materialistic.

The two above codes allowed for the generation of the theme “Mechanisms of Success.”

This acts as a formula and reveals what methods one can use in order to achieve success.

### Extrinsic Factors

“who will define success? It is not me; it is the people who say that you are a success.”

- Participant M3, Medical student, Female

Extrinsic Factors were described by the data as: The external determinants and definition of success perceived by the subject. Be it social, familial or occupational.

### Intrinsic Factors

“it is very difficult to comprehend. You see there are some people who seek happiness of others, others laughing a have a good time is s success. But I think that is the most important success a person you can get. Because you are doing something for mankind.”

### - Participant F1, Faculty, Female

Intrinsic Factors were described by the data as: The subject’s internal organic, comprehension and definition of success.

The two above codes/nodes allowed for the generation of the theme “Concepts of success.” Thus, allowing insight as to how success is defined and perceived, which provides clarity and enables the further understanding thereof. (Table 2)

**Figure 1:**
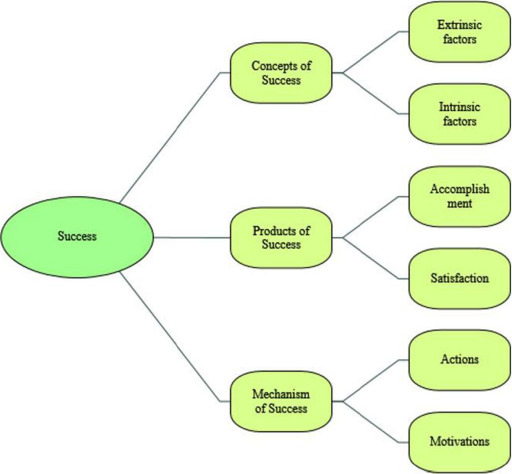
Thematic Analysis

Figure 1 visually represents how the three various themes together form the amalgam which fabricates a roadmap as to how one can attain success by defining the concept, providing a mechanism to achieve it and by highlighting how one can certify that it is truly attained.

## Discussion

### Decoding of Success:

Success is something so intricate, vast and subjective [15]. This study has shed light on its identification and true meaning. The data collected from this study suggests that a quantifiable and tangible demarcation is noticeable between the preconceived general impression of success and the vast multifactorial cohort of intrinsic and extrinsic factors coupled to the highly emotional aspects which were brought forth [16, 17].

### Forms of success:

Researchers have highlighted some noteworthy features whilst classifying success, which are: how the public acknowledges a person’s potential, his gifted or acquired talents and skills, how the significant people surrounding a person react to his success; and the hard work, struggles and attempts made by the individual in order to achieve the goals [18]. A study was performed on the perception of success in adolescents. The majority of adolescents described success as having fame and a name in society. A group research analysis was carried out concerning “success” and the most satisfied group of adolescents correlated success with one’s “personal development and actualization.” They linked success with all the positive feelings such as happiness and personal growth. Another key observation highlighted is that girls who are highly satisfied with their jobs are the ones who eventually become successful [19, 20, 21].

On the contrary, another study conducted on how career-oriented individuals [22, 23] perceive success; suggests that success is being able to achieve one’s personal goals whilst obtaining the expected job satisfaction. They describe job satisfaction as per an individual’s salary, income, bonuses, the products of their labor and another important factor is movement up the corporate ladder. For example, such as receiving work promotions, a raised salary and senior management level positions. A study conducted by Ng et al. 2005 surmised the fact that individuals who have promotions more often and have a greater job satisfaction, are more oriented towards career success [24]. One example is possessing a prominent title or position of power with an organization such as managerial director and or chief executive officer. Career oriented individuals are of the view that success is also coupled to outperforming and surpassing one’s initial expectations, be it personal or organizational. The target is to achieve both personal and occupational goals allowing a career-oriented individual to experience contentment in career growth as well as personal happiness [25]. A fact pertaining to success derived from an opera singer states that she measures success and experiences it by the round of applause that makes her feel elated the end of a performance [26]. The extent to which the views of rich and powerful people influence the lives of others was brought to light by our participants throughout the study. The late great poet laureate, Maya Angelou believes that there is a strong association between success and an individual’s ability to admire himself, to admire what he does and to admire the way he does things. Upon having asked Warren Buffet about his opinion on success, Bill Gates was awed by the former’s answer on how his loved ones’ happiness and the love he derived from them were the sole things which made him happy. On his end, Bill Gates emphasized on how success would be incomplete without invention for the purpose of helping people in need and for raising children in a holistic environment [27].

### Major Themes:

Although the research’s initial aim was to simply understand the “Meaning of success”. The study’s yield surpassed the initial expectations as ultimately the major themes generated were: Products of Success, Mechanisms of Success and Concepts of success. What this produced was a beautiful “recipe” to achieve success, a roadmap if you will. The theme “Concepts of success” was how the data defined success, this is vital as before one can attempt to achieve something it must be understood and well defined. The theme “Mechanisms of Success” provided the insight as to how an individual can achieve success (the steps or practices one can put into place in order to produce a successful outcome.) Finally, the theme of “Products of Success” acts as the marker for achievement, i.e. it is the indicator which proves that success has been attained. The research has therefore proved very effective and has holistically encompassed the “Meaning of success” [9].

### Age and the concatenation of success:

A definite correlation and relationship between age and view of success is apparent. The study was equivocally based on participants younger than 28 years of age and participants older than 55 years of age. As the age of the participants increased there was a definite demarcation in certain views between those of the younger and older participants. The younger participants highly valued the aspect of appeasing older family members and carrying forth pride as their basis of success. Contrary to this the older participants described success on a more holistic basis namely, religion, family well-being, happiness and health [28, 29]. The results very heavily indicated a correlation between the upbringing of participants, their parental influence and childhood. Success also indicated to be birthed from a healthy level of competition between siblings, cousins, friends, fellow colleagues and rivals both on an interpersonal and professional level. This is further supported by evidence from a research published in PloS One 2017 [30].

### Familial dogma and values:

An emphasis was portrayed on family values and lessons taught by elders [31]. Particularly the example set at a young age was vital and enumerated explicitly throughout the interviews. Characteristics which were highly prized and said to be “passed” down from parents or elders were the following: 1) A good work ethic, 2) dedication, 3) perseverance, 4) strategizing, 5) goal setting. The above findings therefore clearly demarcate that family influence has a large and resounding effect on what the participants believe the meaning of success to be and the manner in which it should be approached in order to be achieved. Goal setting, a subtheme which was unequivocally mentioned in the interviews is supported by existing evidence of Blankert T et al. [32].

### Stratagem and success:

It is therefore evident that orchestrating, planning and constructing of time lines is paramount in the achievement of success. “if you fail to plan, you plan to fail” is an extract taken from one of the interviews and this highlights the immense importance of planning in order for one to achieve success. The study brought forth very edifying insights into the minds, psyches as well as beliefs of the participants. There was a definite and striking sense of the close relationship shared between planning and success. The most surprising and unexpected finding allowed for the further continuation of the study whilst simultaneously vastly broadening its preset horizons of its real-world data applications. Success is generally associated with the achievement of a goal or an event which is tangible, however juxtaposed to this the results very evidently and eloquently pinned success to a simple emotion i.e. happiness. A very strong contingency of the participants (90%), described success to be pivoted very heavily on an emotional basis which was associated to an event in time for example, a social gathering, a special birthday celebration or a family event. The above formed the basis of what the participants conceptualize and perceive success to truly be, thus allowing us to have a viewing glass into both the psychosocial and emotional grounding of the study participants. This finding builds on the existing evidence of Lyubomirsky S et al. [15].

### Positivity, persistence and practical implications:

The strong link between positivity and happiness is clearly evident through both this study and the cited study. Positivity and the act of persistence is a vital trait and statistically speaking, favors the attainment of success. The true value of this study is that the results can be used for real world applications. The beauty of “success” is that it is a universally understood term, no matter the level of education or the cultural background of the individual. The practical implication of what this study can yield would be a new, overhauled and fresh personality classification system which would give leaders and people a deeper insight into the psyche and emotional dispositions of their employees or individuals in their immediate surroundings [33]. The establishment of this classification would be groundbreaking in the application of the optimization of human interactions. The simplicity of hearing one’s unbridled view of success could be the key to unlocking a far deeper insight and understanding of that individual, thereby allowing for the optimization of interpersonal dealings and relationship formation [34]. Ultimately maximizing on the storehouse of potential nested in each individual [35].

### Strengths of the study:

This was the first study of such a type to take place in Mauritius, the data collected thus fills the proverbial void of data that is present. The rich information and knowledge gained has staged the setting for further research into this field and the psychology and personality typing through the use of success.

This study was cross sectional in nature in respect to the age of the participants as it gathered information from the young and old alike, this allowing an equilibrium of the findings from both of the age poles to be found.This study was cross sectional in nature in respect to the age of the participants as it gathered information from the young and old alike, this allowing an equilibrium of the findings from both of the age poles to be found.

## Limitation of the study

This study, which was conducted on faculty and medical students from only one medical school in Mauritius

## Conclusion

A quantifiable and tangible demarcation is noticeable between the preconceived general impression of success and the vast multifactorial cohort of intrinsic and extrinsic factors coupled to the highly emotional aspects which were brought forth. The above formed the basis of what students and faculty at SSR Medical College conceptualize and perceive success to truly be, thus allowing us to have a viewing glass into both the psychosocial and emotional grounding of our students and faculty at SSR Medical College.

### Future scope of the study

Recommendations for future progress of this study would be, a mixed method and a multicentric study approach involving medical students and faculty from all the medical schools, which will give a better understanding of the findings.

### What is already known on this topic?

After a thorough literature survey, it was found that the perception of success among adolescents, businessman, career-oriented individuals and clinicians has been reported in the literature.

### What this study adds:

This research forms the basis of what the undergraduate medical students and faculty conceptualize and perceive success to truly be.

## Figures and Tables

**Table 1:** Demographic Characteristics (n=20)

		Faculty (n=10)	Students (n=10)
**Gender**	Male	5(50)	5(50)
	Female	5(50)	5(50)
**Age**	Years	50.10±17.572 years SD	20.80±2.201 years SD
**Nationality**	Indian	6(60)	3(30)
	Mauritian	4(40)	4(40)
	South African	-	3(30)
**Year of study**	First professional	-	2(20)
	Second professional	-	3(30)
	Final Part I	-	2(20)
	Final Part II	-	3(30)

**Table 2:** Code book framework

Node	Description	Example:	Sources	References
**Satisfaction**	The positive emotions and notions intimately related as well as synonymously associated with success.	“laughing and having a good time”	12	23
**Accomplishment**	The attainment and fulfillment of any physical, mental, emotional, social, occupational, personal goal or desire by an individual.	“When I was a commanding officer in the army my job was to become professor of physiology because that was my subject and then if I rise to that level in my subject then I will feel great, and naturally that was success and achievement”	9	15
**Actions**	The arsenal of physical processes, acts of planning, goal setting or forethinking exercised by the individual.	“you keep aspiring and moving in that direction”	6	7
**Motivations**	Drive to attain the preset goal or notion be it positive or negative, applies to factors that enable a subject to strive forwards. Whether it be financial, ultraistic, personal familial or materialistic.	“I think perseverance and self-belief because it is self-belief and self-confidence which gives you positive thinking and if you have positive thinking you will catch onto the opportunity which comes your way and that is what will lead to success.”	11	22
**Extrinsic Factors**	The external determinants and definition of success perceived by the subject. Be it social, familial or occupational.	“who will define success? It is not me, it is the people who say that you are a success.”	4	6
**Intrinsic Factors**	The subject’s internal organic, comprehension and definition of success.	“it is very difficult to comprehend. You see there are some people who seek happiness of others, others laughing a have a good time is s-success. But I think that is the most important success a person you can get. Because you are doing something for mankind.”	9	14
